# Bradykinin, COVID-19, and Pancreatitis, a Personal Perspective

**DOI:** 10.1093/function/zqab046

**Published:** 2021-09-03

**Authors:** Ole H Petersen

**Affiliations:** School of Biosciences, Cardiff University, Cardiff, Wales CF10 3AX, UK

There are individuals you meet early in life who become close friends and remain close friends for your whole life and then there are others you meet early who are more distant, encountered occasionally, but then surprisingly become very important late in your life. For a physiologist, it is the same way with ideas, concepts, systems, and molecules. With regard to molecules, I have had life-long “friendship” with cholecystokinin and acetylcholine, whereas another autopharmacological agent, bradykinin, has been more distant and only recently has become important for my work.

The vasodilator peptide bradykinin was discovered by Mauricio Rocha e Silva and his collaborators, working in Sao Paulo, Brazil, in 1948. The landmark paper reporting the discovery was published the following year in the *American Journal of Physiology*.^1^ Rocha e Silva had worked at the University College London with the great histamine investigator Heinz Schild, whose team included Rod Gregory, who later was my predecessor as George Holt Professor of Physiology at the University of Liverpool. From Rod Gregory (who became famous for isolating and sequencing the peptide hormone gastrin), I heard many stories about the early histamine work and realized that, for too many years, histamine overshadowed bradykinin (and indeed also gastrin).

I first came across the nonapeptide bradykinin when, as an undergraduate clinical student at the University of Copenhagen in the 1960s, I planned experiments on salivary secretion mechanisms and decided to work on the submandibular gland. Starting from scratch, and having no supervisor, I needed to find out how to isolate the submandibular gland, stimulate the nerve activating secretion, and collect the saliva. I found the perfect reference in a paper by Hilton and Lewis,^2^ in which they cited Liddell and Sherrington's beautifully lucid descriptions^3^ of the experiments carried out in the so-called Mammalian Class at the University of Oxford (Nobel Laureate Charles Scott Sherrington regarded the Mammalian Class, which had its origin in his time as George Holt Professor of Physiology at the University of Liverpool, as one of his most important duties in Oxford and, even at the height of his fame and influence, was said never to have missed a single class). One of the classical experiments, conducted in the Mammalian Class, was on the cat submandibular gland, demonstrating that electrical stimulation of the chorda tympany elicited copious fluid secretion and a marked increase in blood flow through the gland.^3^ Having read Hilton and Lewis’ paper,^2^ citing the Liddell and Sherrington experiment, I also took the opportunity to read the second paper by Hilton and Lewis, in that same issue of the *Journal of Physiology*. This second paper^4^ dealt with bradykinin and concluded that “changes in the gland cells, occurring on activation, permit the escape into the interstitial fluid of an intracellular enzyme which acts upon the proteins present to form a vasodilator polypeptide having the pharmacological and physico-chemical properties of bradykinin.” Although this paper^4^ was interesting and convincing, my primary interest was the control of secretion, rather than the control of blood flow, so I did not think much more about bradykinin until, very many years later, my interests became focussed on the pathophysiology of acute pancreatitis. I then took note of an early paper indicating that bradykinin might play an important role in the disease mechanism.^5^

At the time of my renewed interest in bradykinin, around 2014, this small molecule had moved center stage in both physiology and pathophysiology, due to its critical interactions with the renin-angiotensin-aldosterone system and its important role in inflammatory responses, including pain. The kinin system had been recognized as a vital player in blood pressure regulation, together with the renin-angiotensin, natriuretic peptide, and endothelin systems.^6^ The angiotensin converting enzyme (ACE) turned out also to be the enzyme that is mainly responsible for the metabolism of bradykinin. Hence, ACE inhibitors, which are widely used to lower high blood pressure, increase bradykinin levels, and, as bradykinin is a major vasodilator, this augments the antihypertensive effect of ACE inhibitors.^6^

Very recently, the global COVID-19 crisis has brought bradykinin further into the limelight. One of the early studies concerning the action of SARS-CoV-2 showed that the entry of the virus into cells depends on the enzyme ACE2.^7^ Whereas ACE increases blood pressure, partly by lowering the level of bradykinin, ACE2 has the opposite effect. An interesting study, published in the middle of 2020, concluded that SARS-CoV-2 decreased the level of ACE in the lungs, whereas ACE2 increased. The resulting increase in the bradykinin levels in the lungs was termed a bradykinin storm.^8^ It is well known that bradykinin increases vascular permeability, causing fluid extravasation,^6^ and it is therefore plausible that the severe acute respiratory distress syndrome (ARDS), including pulmonary edema, seen in severe cases of COVID-19, is caused at least in part by the bradykinin storm.^8^ In this context, it is interesting to note that one of the well-known side effects of treating hypertension with ACE inhibitors is persistent cough due to increased bradykinin action.^6^ Persistent cough is of course one of the cardinal symptoms of COVID-19.

From my personal and pancreatic perspective, it was intriguing that severe acute pancreatitis can also result in ARDS. Furthermore, it had been known for a long time that plasma bradykinin levels are elevated in pancreatitis.^9^ At least part of the reason for this is likely to be release from necrotic acinar cells of kallikrein, the enzyme that is responsible for catalyzing the formation of bradykinin.^9^ Bradykinin evokes prominent Ca^2+^ signals in the peri-acinar stellate cells, even at concentrations that are only slightly higher than those in plasma from healthy resting animals or humans. The steepest rise of the concentration-response curve occurs between the normal plasma level of bradykinin and the concentration observed in patients with acute pancreatitis. Bradykinin also evokes Ca^2+^ signals in the pancreatic macrophages, albeit at slightly higher concentrations.^9^ Recent evidence suggests that bradykinin-elicited Ca^2+^ signals mediate secretion of substances, including cytokines, that drive necrotic amplification loops between acinar, stellate, and immune cells in the exocrine pancreas.^9^ The cytokine and bradykinin storms initiated in the pancreas in severe cases of acute pancreatitis have body-wide implications potentially causing multiorgan failure, including ARDS.

It is still too early to predict whether pharmacological interventions aimed at reducing either bradykinin formation or its actions will turn out to be helpful in the treatments of COVID-19 and/or acute pancreatitis. Given that bradykinin was discovered by Brazilian scientists,^1^ it seems natural that a clinical trial, evaluating the efficacy and safety of a bradykinin (type 2) receptor antagonist (icatibant) and a C1 esterase/kallikrein inhibitor, is now in progress in Brazil.^10^

If bradykinin turns out be central to the development of COVID-19 as well as acute pancreatitis, and treatments to reduce bradykinin levels or its effects are found effective in combatting these often devastating diseases, then this will be a truly remarkable story, in which a molecule that for a long time was overshadowed by histamine, and thought by many to be of relatively minor interest, suddenly takes center stage in the fight against a major pandemic.
